# Remote Home Monitoring of Older Surgical Cancer Patients: Perspective on Study Implementation and Feasibility

**DOI:** 10.1245/s10434-020-08705-1

**Published:** 2020-06-29

**Authors:** Leonie T. Jonker, Matthijs Plas, Geertruida H. de Bock, Erik Buskens, Barbara L. van Leeuwen, Maarten M. H. Lahr

**Affiliations:** 1 Department of Surgical Oncology, University of Groningen, University Medical Center Groningen, Groningen, The Netherlands; 2 Department of Epidemiology, University of Groningen, University Medical Center Groningen, Groningen, The Netherlands

## Abstract

**Background:**

Remote home monitoring might fill the perceived surveillance gap after hospital discharge. However, it is unclear whether older oncologic patients will be able to use the required new digital technologies. The study aimed to assess the feasibility of postoperative remote home monitoring for this population.

**Methods:**

This observational cohort study recruited patients aged 65 years or older scheduled for oncologic surgery. The study patients used a mobile application and activity tracker preoperatively until 3 months postoperatively. A subset of the patients used additional devices (thermometer, blood pressure monitor, weight scale) and completed electronic health questionnaires 2 weeks after hospital discharge. Feasibility was assessed by the study completion rate, compliance in using components of the information technology system, acceptability [Net Promotor Score (NPS)] and usability [System Usability Scale (SUS)]. The NPS score varied from − 100 to + 100. An SUS higher than 68 was considered above average.

**Results:**

Of 47 participants (mean age, 72 years; range, 65–85 years), 37 completed a follow-up assessment, yielding a completion rate of 79%. Compliance in using the activity tracker (*n* = 41) occurred a median of 81 days [interquartile range (IQR), 70–90 days] out of 90 post-discharge days. Compliance in measuring vital signs and completing health questionnaires varied from a median of 10.5 days (IQR, 4.5–14.0 days) to 12 days (IQR, 5–14 days) out of 14 days. The NPS was + 29.7%, and the mean SUS was 74.4 ± 19.3.

**Conclusion:**

Older oncologic patients in the study considered postoperative home monitoring acceptable and usable. Once they consented to participate, the patients were compliant, and the completion rate was high.

**Electronic supplementary material:**

The online version of this article (10.1245/s10434-020-08705-1) contains supplementary material, which is available to authorized users.

The increasing incidence of cancer in patients older than 65 years is a global challenge.^1^ In 2018, cancer was newly diagnosed for 7.2 million older patients worldwide, excluding non-melanoma skin cancer.^2^ This number is predicted to increase to 14 million by 2035, representing 60% of the total cancer cases.^1^

Because surgery is essential in more than 80% of new cancer cases, the total number of patients demanding surgery will be approximately 17.3 million by 2030.^3^ Therefore, the percentage of onco-geriatric patients presenting for surgery as part of oncologic treatment also will continue to increase.^3^

Modern health care changes in postoperative care management have led to considerably shortened hospital admissions, especially in high-income countries.^4^ Notably, once patients have been discharged, the extent and intensity of guidance and monitoring of recovery is limited, whereas the days after hospital discharge are a vulnerable period.^5^ More than half of onco-geriatric patients experience at least one complication within 30 days after surgery.^6^ Postoperative complications occur more frequently after the patient has left the hospital, partly due to earlier hospital discharge.^7^,^8^ To avoid more invasive treatment of complications or even readmission (i.e., to reduce medical consumption and health care costs and improve clinical outcomes), timely recognition and management of deviations in recovery are of the utmost importance.^9^

A useful tool to bridge the reported gap in guidance and monitoring after hospital discharge could be the use of eHealth, defined as “health services and information delivered or enhanced through the Internet and related technologies,”^10^ could be a useful tool to bridge the reported gap in guidance and monitoring after hospital discharge. Remote home monitoring of the postoperative recovery experienced by older patients using eHealth has been described mainly in cardiac and orthopedic surgery,^11^,^12^ but has scarcely been studied in onco-geriatric surgery.^13^ New digital technologies can potentially detect complications early and prevent unplanned readmissions. However, it remains unclear whether and under which conditions older oncologic patients will be able to use these technologies.

Therefore, this study aimed to investigate the process of implementing a novel information technology (IT)-supported integrated care management system using a mobile application and additional smart devices for remote home monitoring of older patients after oncologic surgery.

## Methods

### Context

In high-income countries, each patient is generally evaluated to determine the need for extra care after hospital discharge. In case extra care is needed, this often is arranged via home care services or by referral of the patient to a nursing home/rehabilitation center. In the Netherlands, patients can contact the hospital for questions during the first days after hospital discharge, but the general practitioner is the first point of contact for the patients once they are discharged to their home.^14^ A follow-up consultation with the surgeon is scheduled several weeks after surgery in most cases. In case of postoperative care, including diagnosis and treatment of complications, the costs are reimbursed by health insurance companies.

### Development of Connecare

An IT-supported care management system aimed at integrating care services for people with chronic long-term conditions was developed within the Connecare consortium, funded by the European Union’s Horizon 2020 Research & Innovation Program (project grant agreement no. 689802).^15^ Several European technical and clinical partners co-designed an IT system (Connecare) that consisted of two components: (1) a Smart Adaptive Case Management System (SACM), a web-based professional interface used by researchers and professionals, and (2) a Self-Management System (SMS), an application for patients’ use. Clinical partners in three European countries used a customized version of the IT system adapted to the local context and a clinical trial corresponding to their specific needs and aims.

### Study Design and Participants: Local Study Implementation of Connecare

This was a single-center observational feasibility study with gradual implementation of eHealth tools for remote home monitoring of older patients following their hospital discharge after oncologic surgery. This study was approved by the local medical ethics committee (registration no. 2017/286; Netherlands trial registration no. NL8253).

The inclusion criteria specified patients older than 65 years with elective oncologic resection of a solid tumor in the department of oncologic surgery and gynecology at a tertiary referral center in the Netherlands, Internet access at home, and written consent. The exclusion criteria ruled out emergency surgical intervention; severe visual, hearing, or cognitive impairment; insufficient understanding of the Dutch language; and cancellation of surgery.

### Connecare Remote Home Monitoring System

The components of the Connecare Remote Home Monitoring System are listed in Table 1. Because integration of additional smart devices with the Connecare system still was under development at the beginning of the study, we started monitoring with the first available monitoring tool, a Fitbit activity tracker (Fitbit Inc., San Francisco, CA, USA), which measured physical activity. Additional tools for remote home monitoring of other vital signs and patient-reported symptoms were introduced in a stepwise fashion when integration with the IT management system was actualized (Table 1). This also gave us the possibility to test and further develop the system during study implementation, with IT support still available for the Connecare project. We distinguished an “early” cohort of patients who used a subset of the monitoring system and a “late” cohort of patients who used the complete monitoring system including all the smart devices and electronic questionnaires.

**Table 1:** Components of the Connecare remote home monitoring system

*I. Smart Adaptive Case Management System (SACM)* Professional website used by the case manager	
Case manager enables the monitoring of physical activity, vital signs measurements, and/or electronic health questionnaires Possibility to monitor patients’ real-time health data Alert system alarms when value is outside preset range A screenshot of the SACM is provided in Fig. S1	
*II. Self*-*Management System (SMS)* Application for patients’ use	
Pre-installed on patient’s smartphone or study tablet (ASUS ZenPad 10, ©ASUSTeK Computer Inc., Taipei, Taiwan and Samsung Galaxy Tab A, Samsung, Seoul, South Korea) Possibility to connect to various smart devices (see later) for measurements Demonstrates postoperative recovery to the patients A screenshot of the SMS is provided in Fig. 2	
*III. Connected smart devices* Commercially available monitoring devices connected to Connecare system	
Connected via commercially available applications on smartphone/tablet Smart device applications were connected to the SMS Data were automatically transferred from the smart device application to the SMS and SACM	
a).	Activity tracker (Fitbit Charge 2; Fitbit Inc., San Francisco, CA, USA)
Connects with Fitbit application Alarm settings step count < 1000 (when average of steps normally is > 1000 steps) Implemented from May 2018	
b).	Thermometer (Thermo; Nokia Withings, Issy-les-Moulineaux, France)
Connects with thermo application Alarm settings temperature < 36 °C or > 38 °C Implemented from October 2018	
c).	Blood pressure monitor (BPM; Nokia Withings, Issy-les-Moulineaux, France)
Connects with Health Mate application Alarm settings blood pressure < 100/60 mmHg or > 150/100 mmHg Alarm settings heart rate < 50/min or > 100/min Implemented from November 2018	
d).	Weight scale (Body + , Nokia Withings, Issy-les-Moulineaux, France)
Health Mate application Alarm setting weight: − 5% or + 5% of weight at hospital discharge Implemented from December 2018	
*IV. Electronic health questionnaires* Electronic questionnaires with postoperative patient-reported symptoms	
Translated into Dutch Created in the SACM Available for answering by the patient in SMS Answers visible for patient in SMS and for case manager in SACM	
a).	Pain questionnaire (Visual Analogue Scale, linked to a Numerical Rating Scale with 0 being “no pain” and 10 being “the worst pain imaginable”)
Alarm setting: score higher than on previous day Implemented from December 2018	
b).	Postsurgical health questionnaire (patient-reported symptoms)
12 Yes/no-choices that asks for problems regarding (1) breathing, (2) vomiting, (3) dizziness, (4) eating, (5) drinking, (6) urinating, (7) defecating, (8) mobility, (9) fever, (10) resting and sleeping, (11) bathing and washing, (12) getting (un)dressed Alarm setting in case of problems with breathing, vomiting, dizziness or fever Implemented from December 2018	

### Study Procedure

The case manager approached eligible patients for study participation in chronological order of diagnosis before scheduled surgery face-to-face at the outpatient clinic or by telephone. A baseline assessment was performed 1 to 4 weeks before surgery at home or during a visit in the outpatient clinic.

The case manager instructed the patients how to use the applications and the activity tracker. The patients wore the activity tracker on their wrist preoperatively to determine the baseline step count and postoperatively in the surgical ward, then at home until 3 months after surgery. During surgery and intensive care unit (ICU) admissions, the patients did not wear the activity tracker. No step goal was provided.

Before hospital discharge, some of the patients received additional smart devices (e.g., thermometer, blood pressure monitor, or weight scale) and instructions on how to check their vitals with the devices once per day during the first 14 days after hospital discharge. The patients were instructed to contact the surgical nurse or their family physician if they noticed any deviation in their recovery.

**Fig. 1:**
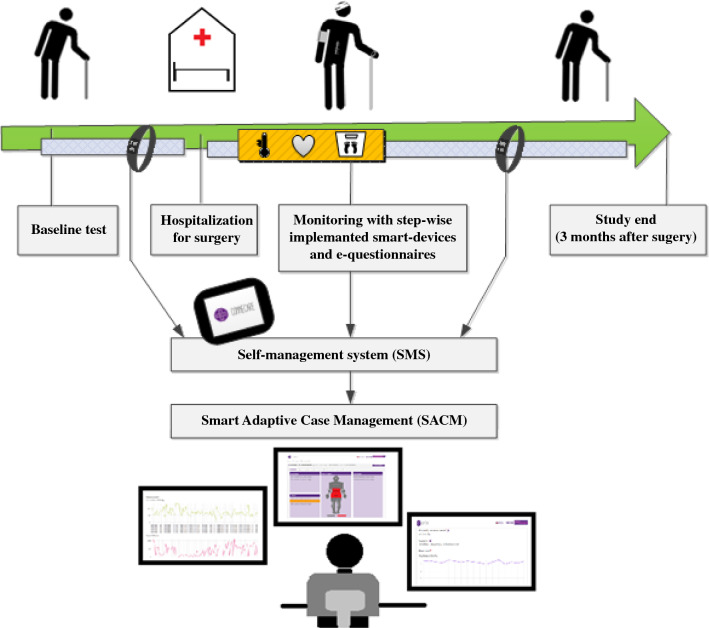
Infographic depicting the Connecare IT system and study logistics

Data were not real-time monitored but checked daily during weekdays. If alarming parameters were present during this data check (listed in Table 1), they were interpreted and analyzed by the case manager (physician). If the case manager did not receive the data or observed abnormal findings, the patient was contacted by telephone for additional information. The treating physician remained available to discuss further actions, and the monitoring with the smart devices would be extended for 14 days if a complication was detected. Data collected by smart devices were securely stored in a server provided by Eurecat S.A. (Barcelona, Spain). Data were handled confidentially and anonymously in compliance with the Dutch Personal Data Protection Act. Figure 1 illustrates the study logistics.

### Collection of Baseline Data

Demographics and baseline characteristics were collected from medical records including comorbidity (Charlson Comorbidity Index^16^) and preoperative physical status classification by an anesthesiologist (American Society of Anesthesiologists^17^). Other characteristics assessed at baseline were frailty (measured using the Groningen Frailty Indicator^18^), functional performance ([instrumental] Activities of Daily Living^19^,^20^), nutritional status (short-form mini-nutritional assessment^21^), physical performance (Timed Up&Go^22^), hand grip strength^23^), mental well-being (Hospital Anxiety and Depression Scale^24^,^25^), and self-reported physical activity (Short QUestionnaire to ASses Health-enhancing physical activities^26^[SQUASH]).

### Feasibility of Connecare

To evaluate the feasibility of Connecare, the patients had to complete usability and acceptability questionnaires (on paper) at follow-up assessment 3 months after surgery. Usability was assessed by the System Usability Scale (SUS), a questionnaire consisting of 10 statements regarding the usability of an electronic device or system that participants can rate on a 5-point Likert scale.^27^ The Connecare system was considered usable if the mean SUS was higher than 68.^28^,^29^

Acceptability was assessed with a satisfaction questionnaire that asked patients about their general impressions, the user-friendliness of the system, their ability to use the system without help, and their Net Promotor Score (NPS).^30^ Using a scale of 0 to 10, the NPS is calculated based on responses to the question: *“*How likely is it that you would recommend our company/product/service to a friend or colleague?” The percentage of detractors (answering with 0 to 6) was subtracted from the percentage of promoters (answering with 9 to 10). Scores of the passives (answering with 7 or 8) were not counted. An NPS could be as low as − 100 or as high as + 100. A positive total NPS was considered acceptable.^30^

Additionally, we asked participants whether they synchronized the Fitbit and measured vital signs independently, whether they were helped by partners or children, or whether partners or children performed the tasks for them. Other feasibility metrics included the study completion rate (% of participants who completed the follow-up assessments) and compliance. Compliance with the use of the post-discharge remote home monitoring system included the activity tracker (number and % of the 90 postoperative days that a daily step count > 0 was transferred to the SACM), smart devices (number and % of the 14 days that vital signs were transferred to the SACM), and electronic health questionnaires (number and % of the 14 days that questionnaires were completed and transferred to the SACM).

Variability in monitored parameters was divided into inter-subject variability (average variability between subjects at one measurement moment in time) and intra-subject variability (average variability in one patient over time). No cutoff values for these feasibility metrics have been previously established. However, based on previous postoperative telemonitoring studies, we considered feasibility to be indicated by a completion rate higher than 65% to 75%,^31^,^32^ a compliance rate higher than 67% for synchronization of physical activity data,^32^ and a rate higher than 85% for vital signs measurements.^32^ Reasons for ineligibility and decisions not to participate in the study or to drop out were assessed by the case manager and prospectively registered in the database. Newly encountered logistical problems as well as the solutions implemented during the study and data collection were documented by the research team in a log.

### Outcomes

The following four outcome measures were used: participation rate (% of eligible patients willing to participate in the study), reasons for declining participation, logistic problems encountered, their solutions, and feasibility. The feasibility metrics were completion rate, compliance, usability, and acceptability.

### Statistical Analysis

Patient characteristics and study outcomes were summarized using descriptive statistics, and comparisons between patients were performed using parametric or nonparametric tests. For the sake of illustration, means of SUS and acceptability scores were presented instead of medians, and *p* values were based on nonparametric testing. To compare the subgroup of patients who found the system not usable with the subgroup of patients who found it usable, subgroups were created based on the SUS score (SUS < 68 vs ≥ 68). To compare the patients who considered the system not acceptable with the patients who considered it acceptable, subgroups were created based on the response to the question: “How likely is it that you would recommend our company/product/service to a friend or colleague?” (detractors vs passives and promotors). Inter- and intrasubject variability in measured parameters was presented using the median value with the interquartile range (IQR). The correlation between the preoperative activity reported by SQUASH and the data collected was calculated using Spearman’s rank correlation coefficient. Data were analyzed with IBM SPSS Statistics version 23 (IBM Corporation, Armonk, NY, USA).

## Results

### Recruitment and Nonparticipants

**Fig. 2:**
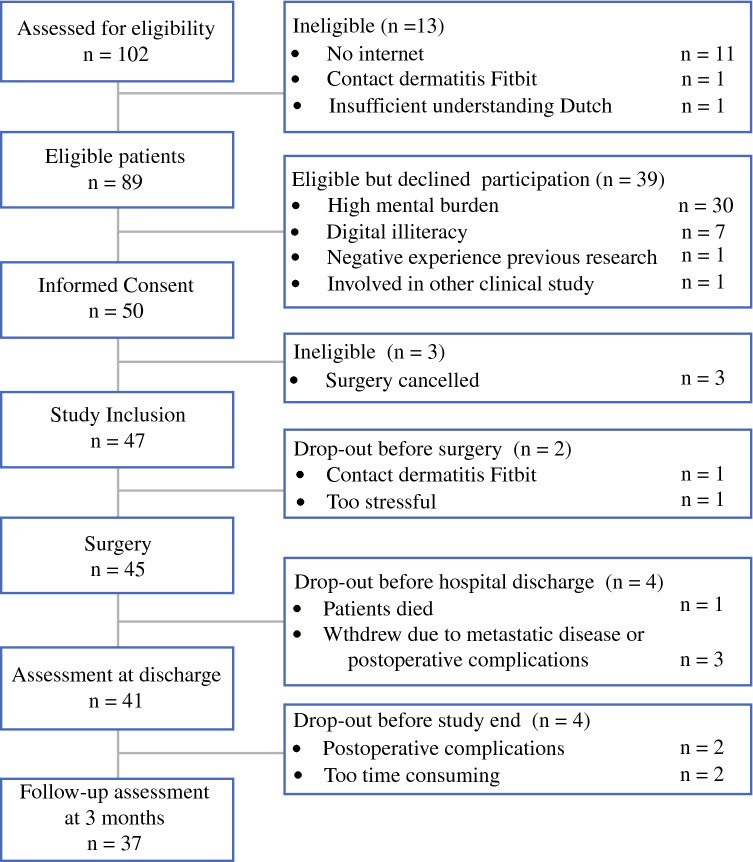
Study flowchart

From May 2018 until June 2019, 102 patients were informed about the study (Fig. 2). Of 89 eligible patients, 50 consented to participate, yielding a participation rate of 56%. The main reason for patients to decline study participation was a perceived high mental burden in a time of stress for surgery (*n* = 30, 77%). The patients who declined were more often female (56% vs 32%; *p* = 0.018) and older (mean age, 76 ± 5.8 vs 73 ± 5.4 years; *p* = 0.009) than the participating patients. Three patients who consented to participate became ineligible because their surgical procedures were cancelled due to a high risk of perioperative morbidity and mortality. These patients were therefore excluded, and analysis was performed with 47 participating patients, 23 patients in the early cohort (May 2018 to November 2018) and 24 patients in the late cohort (January 2019 to June 2019).

### Sociodemographic and Clinical Characteristics of the Participants

The 47 participating patients had a mean age of 72.2 ± 5.0 years (range, 65–85 years), and 31 (66%) were male. Table 2 presents the patient characteristics and results of the baseline assessment. One patient decided to leave the study before completing the baseline assessment because of dermatitis related to wearing the activity tracker. Other decisions of patients to drop out are listed in the study flowchart depicted in Fig. 2. The 41 patients who remained in the study at hospital discharge used the activity tracker for postoperative assessment at home.

**Table 2:** Patient characteristics

Variables (*n* = 47)	*n* (%)
*Age (years)*	
65–70	18 (38.3)
71–75	18 (38.3)
76–80	7 (14.9)
> 80	4 (8.5)
*Gender*	
Male	31 (66)
Female	16 (34)
*Nationality*	
Dutch	47 (100)
*Housing*	
Independent, alone	10 (21)
Independent, with others	37 (79)
*Highest level of education*	
Primary school	4 (9)
Secondary school	23 (49)
Secondary vocational school	11 (23)
Higher education/university	9 (19)
*Current employment (yes)*	5 (10.6)
*Use of electronic devices at home*	
Smartphone and/or tablet	40 (85.1)
*ASA classification*	
1	3 (6)
2	35 (75)
3	9 (19)
*Charlson comorbidity index: median (IQR)*	4 (2–6)
*Tumor location*	
Intracavitary	37 (79)
Superficial	10 (21)
*Baseline assessment (n *=* 46)*	
Frail (GFI > 4)	5 (11)
Impaired ADL (ADL ≥ 5)	0 (0)
Impaired iADL (iADL ≤ 7)	12 (26)
Mental status: anxiety (HADS-A ≥ 7)	4 (9)
Mental status: depression (HADS-D ≥ 5)	16 (35)
Risk of malnutrition (MNA-SF ≤ 11)	14 (30)
Slow-timed Up&Go (> 12 s)^a^	2 (5)
Low muscle strength (handgrip strength)^b^	7 (17)
Low subjective moderate–vigorous physical activity (SQUASH < 150 min/week)^c^	24 (63)

*ASA* American Society of Anesthesiologists, *IQR* interquartile range, *GFI* Groningen Frailty Indicator,^18^*ADL* activities of daily living,^19^*iADL* instrumental activities of daily living,^20^*HADS*-*A* Hospital Anxiety and Depression Scale–Anxiety,^24^,^25^*HADS*-*D* Hospital Anxiety and Depression Scale–Depression,^24^,^25^*MNA*-*SF* Mini Nutritional Assessment–short form^21^

^a^Timed Up&Go^22^ was performed by 44 patients

^b^Hand grip strength^23^ was assessed in 42 patients

^c^SQUASH: Short QUestionnaire to ASses Health enhancing physical activity,^26^ assessed in 38 patients

In the stepwise implementation of smart devices, the thermometer was the first supplementary smart device used for postoperative remote monitoring of patients after validation, updating, and testing of the IT system, followed by the other smart devices and electronic health questionnaires (Table 1).

### Feasibility Metrics

**Fig. 3:**
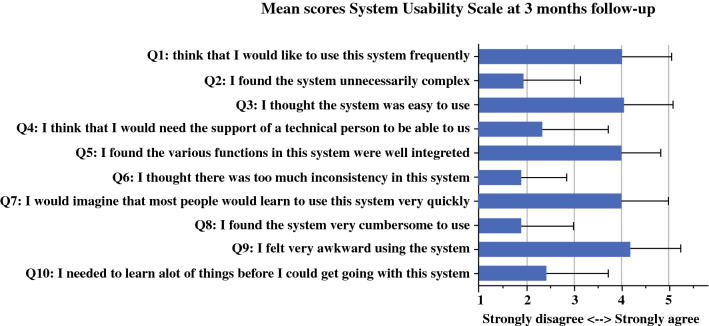
Mean scores of the system usability scale at the 3-month follow-up assessment (*n* = 37)

**Fig. 4:**
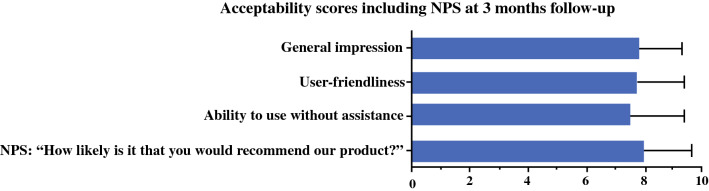
Mean acceptability scores, including the score at the 3-month follow-up assessment (*n *= 37). NPS, Net Promotor Score

Of the 47 study patients, 37 completed the study follow-up assessment, resulting in a completion rate of 79% (37/47). The compliance rates for postoperative wearing and synchronization of the activity tracker and for performing measurements with other smart devices and answering electronic health questionnaires varied between 75 and 87%, as illustrated in Table 3. Overall usability and acceptability scores are presented in Figs. 3 and 4. In the early cohort, the mean score was 73.1 ± 15.1 on the SUS (range, 47.5–97.5) and +29.4% on the NPS. In the late cohort, the mean score was 75.5 ± 22.6 (range, 22.5–100.0) on the SUS and +30.0% on the NPS. The 13 patients who considered the usability of the system poor had a lower level of education than the 24 patients who considered it usable (*p* = 0.02). Also, the patients with a low usability score were older (mean age, 74.5 ± 4.5 vs 71.9 ± 5.2 years; *p* = 0.15), more often female (46% vs 30%; *p* = 0.46), more frequently living alone (39% vs 17%; *p* = 0.23), and more frequently using their own tablet at home (46% vs 77%; *p* = 0.08) than the patients with a high usability score, although these differences were statistically not significant. The 5 patients who did not find the system acceptable were comparable in age, gender, housing, education level, and use of electronic devices at home with the 32 patients who considered the system acceptable. Four of the five detractors considered the system also not usable.

**Table 3:** Compliance with the use of devices in remote home monitoring system

Parameter	Patients (*n*)	Duration of monitoring (days)	Days with measurements median (IQR)	Compliance in % median (IQR)
Physical activity	41	90	81 (70–90)	90.0 (77.8–100.0)
Temperature	30	14	10.5 (5.8–13.0)	75.0 (41.1–92.9)
Blood pressure	29	14	12.0 (5.0–14.0)	85.0 (35.7–100.0)
Heart rate	29	14	11.0 (2.5–14.0)	78.6 (17.9–100.0)
Weight	25	14	11.0 (2.5–12.0)	78.6 (32.1–100.0)
Questionnaire (pain)	24	14	11.0 (4.5–14.0)	78.6 (32.1–100.0)
Questionnaire (postsurgical patient-reported symptoms)	24	14	10.5 (4.5–14.0)	75.0 (32.1–100.0)

*IQR* interquartile range

Variability differed per measured parameter. A low variability was observed in temperature measurements in both the inter-subject analysis (median, 36.5 °C; IQR, 0.3 °C) and the intra-subject analysis (median, 36.6 °C; IQR, 0.4 °C). A larger variability was observed in blood pressure and heart rate measurements, with comparable inter- and intra-subject variability [median systolic blood pressure: 126 mmHg (IQR, 12.9 mmHg) vs 128 mmHg (IQR, 11.9 mmHg); median diastolic blood pressure: 72 mmHg (IQR, 9.0 mmHg) vs 73 mmHg (IQR, 7.6 mmHg), and heart rate: 71.3 bpm (IQR, 10.4 bpm)].

The inter-subject variability of weight was higher than the intra-subject variability [79 kg (IQR, 8.3 kg) vs 80 kg (IQR, 1.0 kg)]. The variability in preoperative step count was large, with a larger variability in the inter-subject step count [median, 5392 steps (IQR, 5446 steps)] than in the intra-subject step count [median, 6567 steps (IQR, 3932 steps)]. The reported preoperative activity by SQUASH had a moderate positive correlation with the preoperative step count (Spearman’s rho, 0.42; *p* = 0.016).

### Logistical Problems Encountered and Their Solutions

During the first months of the study the rate of patient inclusion in the study was low. To solve this, eligible patients were contacted mostly face-to-face at the outpatient clinic instead of being approached by telephone. This resulted in an increased participation rate, from 33% (6/18) in May through September 2018 to 63% (44/70) from October through June 2019.

The patients experienced usability problems (e.g., text and icons too small) and excessive mobile data usage due to continuous synchronization of steps to the application when using the application on their own smartphone. In addition, a number of patients had old smartphones not suitable to run the application. Finally, some patients interested in participating did not use a smartphone at all. To solve these issues, we provided all the patients with tablets from June 2018 until the end of the study.

Some of the participants encountered difficulties connecting new devices to their home Wi-Fi networks and reproducing instructions given to them at preoperative assessment. Because of this, preoperative assessment, instruction, and connecting to Wi-Fi preferably took place at patients’ homes (*n* = 43) rather than at the hospital. Considerable delays in the data transfer from the thermometer and weight scale to the applications via Wi-Fi were solved through connecting via Bluetooth instead (thermometer). The patients concluded that it was easier to enter their weight into the Connecare application manually than to use the smart weight scale.

Logging into the SMS with patients’ own email addresses and chosen passwords appeared to be time-consuming at installation. The login information was hard for the patients to remember. Therefore, we preinstalled all applications on the tablets and created user names and passwords for study purposes.

The patients had more difficulty than expected in the synchronizing of the Fitbit data. Consequently, a pamphlet with basic user information was added to the baseline instruction from October 2018. The instructions were adjusted based on previous reported usability problems of patients. When patients called the case manager with usability issues, the explanation proved to be more effective when they were referred to the paper instruction pamphlet at the same time.

Synchronization of Fitbit data was performed by 34 patients (83%) without help from partners or children. Only 21 (72%) of the patients completed the vital signs measurements and electronic questionnaires without help. Consequently, we intended to provide instructions about post-discharge monitoring in attendance of a family member. Supplementary Textbox S3 provides an overview of the most important lessons learned from the logistical problems encountered during this study.

## Discussion

### Principal Findings

This report describes the stepwise study implementation process and feasibility of a novel IT system using smart devices for home monitoring of older patients after oncologic surgery. Remote home monitoring was considered feasible, usable, and acceptable by the older patients who participated in this study, as measured on the usability (SUS) and acceptability (NPS) scales. Once the patients consented to participate, they were compliant in the wearing and synchronizing of physical activity tracker and with some vital sign measurement, and the completion rate was high.

### Comparison with Prior Work

This study is one of the few that has reported on the development, study implementation, and clinical feasibility of a novel IT system for remote home monitoring of older oncologic patients, a population often left out of clinical trials.^33^ Wynter-Blyth^34^ concluded that remote home monitoring of nine patients (median age, 70 years) with esophago-gastric cancer using an eHealth application, activity tracker, wireless finger probe, and weight scale was feasible. However, they used qualitative patient feedback as well as unspecified usability and acceptability questionnaires rather than the validated questionnaires (SUS and NPS).^27^,^30^ Metcalf et al.^32^ determined the feasibility of their health care application for 20 patients (median age, 70 years) after radical cystectomy based on the high compliance rate. Our compliance with wearing and synchronizing of the activity tracker was higher, but overall compliance in measuring vital signs was lower than in the Metcalf et al.^32^ study.

Our study’s participation rate of 57% was lower than in studies with less complex eHealth interventions for older cancer patients.^31^,^35^ Consistent with our results, Skender et al.^35^ noted that patients who refused participation were significantly older than patients who participated. It is known that patients with low health literacy are less willing to participate in cancer trials.^36^ In addition, eHealth applications for self-management are less likely to be used by older, unmarried, less educated, unemployed, and lower-income cancer survivors.^37^ For this reason, we must make sure that new eHealth interventions do not further increase the gap between high and low health literacy patients regarding their health outcomes.

### Study Strengths and Limitations

A strength of our study was the commitment of the patients who used the remote monitoring system after hospital discharge until the end of the study. A completion rate of 79% (37/47) means that 21% discontinued home monitoring. It is troublesome that postoperative course data are missing for the patients who experienced complications or considered the measurements too time-consuming because this population is of particular interest for monitoring and might benefit most from early detection of complications. However, of the 41 patients discharged from the hospital with monitoring, 37 (90%) wore the activity tracker for more than 90% of the days before the 3-month follow-up evaluation and completed usability and satisfaction questionnaires. Moreover, comparable or lower completion rates have been reported in telemonitoring studies with older surgical patients.^31^,^32^,^38^

Both an advantage and a limitation to our study was that the study started with the IT system still under development. Because of a fixed end date for the project and corresponding IT support, it was not possible to wait to include patients until the latest version of the application was available. This resulted in a stepwise introduction of smart devices. The advantage was the opportunity to solve usability and logistical problems in phases before introduction of the next smart device to other patients. A limitation was that usability and acceptability scores were completed by patients whose experience of the remote home monitoring system differed with respect to the number of smart devices used, although usability and acceptability scores were comparable between the late and early cohorts. In addition, an important limitation of this study was the observational study design without interventions based on the results of real-time data monitoring.

### Clinical Implementation and Future Perspectives

We anticipate that the results of this study will facilitate others in overcoming barriers in future studies. However, before remote home monitoring of older patients can be used after cancer surgery outside a study setting, further research is required on several aspects of remote home monitoring.

First, recommendations for optimal postoperative home monitoring in this population are required. Within the population of onco-geriatric patients, most post-discharge complications and unplanned readmissions of older patients after cancer surgery are reported to result from infections or cardiovascular causes, immobility, or malnutrition.^7^,^39^,^40^ Therefore, postoperative remote home monitoring systems for older cancer patients not only measured various vital signs with a high predictive value for hospitalization,^41^,^42^ but also were able to detect immobilization and weight loss. Oxygen saturation measured in other remote home monitoring studies of older patients,^32^,^34^ could be a valuable addition to this system.

Second, a study should investigate how remote home monitoring could be integrated into an existing health care system. To gain further insight into feasibility, a qualitative assessment of wishes, needs, and ideas from older patients with cancer and their health care professionals could be of additional value. This would further promote professional engagement and acceptability of actual implementation of a novel IT system in clinical practice.

In future studies, predictive parameters for complications and unplanned readmissions after oncologic surgery should be identified. It would be challenging to develop a home monitoring system that comprehensively captures a wide range of parameters and still is easy for older patients to use. Promising single-monitor devices that capture various parameters have already been studied for use in the hospital setting and could also help improve usability and patients’ compliance with remote home monitoring in the future.^43^

## Conclusion

A novel IT system to monitor older patients after oncologic surgery was successfully developed, and subsequently implemented. The patients found postoperative home monitoring feasible, acceptable, and usable in the study setting. Once they consented to participate, patients were compliant with regard to wearing and synchronizing the physical activity tracker, and the completion rate was high. The compliance rates for measurement of vital signs and completion of health questionnaires were lower but acceptable. Future studies should evaluate trends in vital parameters of home monitoring, identify predictive home monitoring parameters for postoperative complications and unplanned readmissions, and explore integration into the existing health care system.

## Electronic supplementary material

Below is the link to the electronic supplementary material.

Supplementary material 1 (DOCX 207 kb)
