# Supplementary material for: Remote Home Monitoring of Older Surgical Cancer Patients: Perspective on Study Implementation and Feasibility
Source: Ann Surg Oncol. 2020 Jun 29;28(1):67–78. doi: 10.1245/s10434-020-08705-1 (PMC7752881; doi:10.1245/s10434-020-08705-1)
Supplement: Supplementary file 1 — Supplementary material 1 (DOCX 207 kb) [file 10434_2020_8705_MOESM1_ESM.docx]

## Supplemental material

**Supplementary Figure S1.** Screenshot of the Smart-Adaptive Case Management System

**
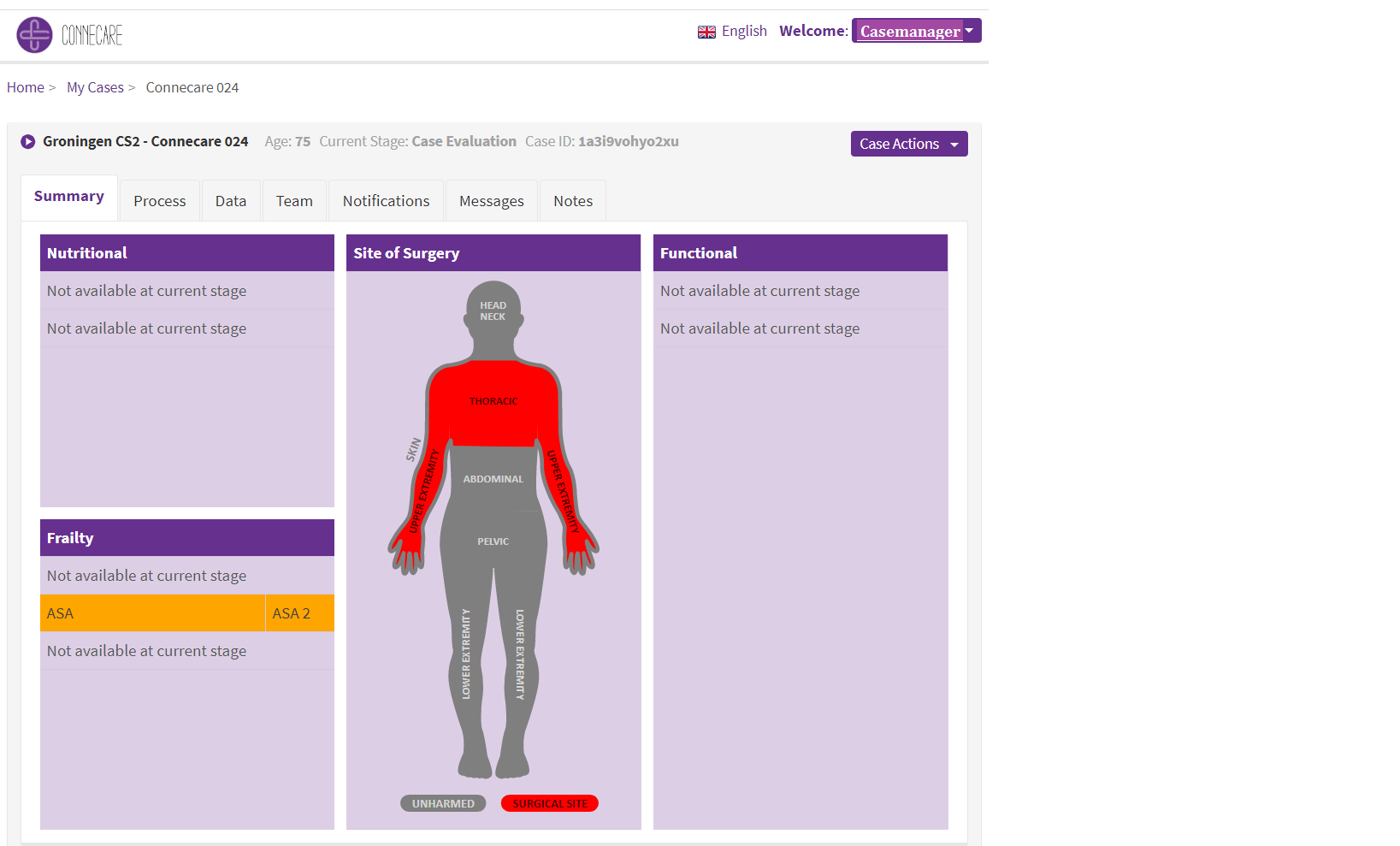
**

**Supplementary Figure S2.** Screenshot of the Self-Management System

**
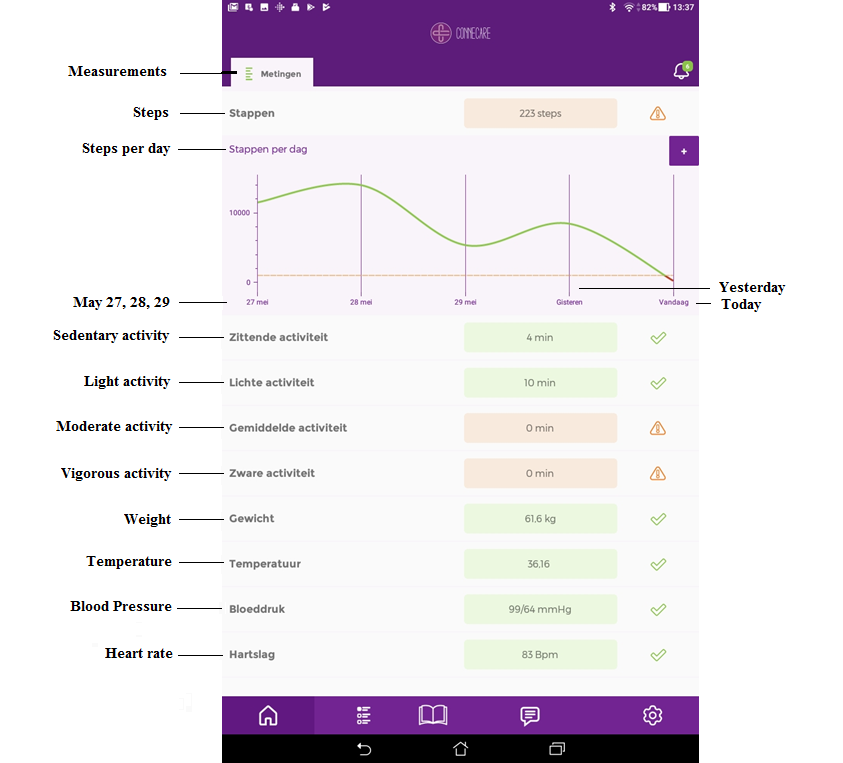
**

| **Supplementary Textbox S3:** Lessons learned from logistical problems encountered during a telemonitoring study in older surgical patients |
| --- |
| To increase participation rate:   - Approach patients face-to-face to increase participation rate   To increase acceptability:   - Provide instructions in a familiar environment* - Involve family members when providing instructions^†^ - Provide instructions on paper   To increase usability:   - Use tablets instead of smartphone - Pre-install applications on tablet - Pre-install tablet and devices on WiFi or Bluetooth network - Offer support by telephone if usability issues occur - Involve end-users early in the study implementation phase |
